# InChI in the wild: an assessment of InChIKey searching in Google

**DOI:** 10.1186/1758-2946-5-10

**Published:** 2013-02-11

**Authors:** Christopher Southan

**Affiliations:** 1 TW2Informatics, Göteborg 42166, Sweden

**Keywords:** InChI, InChIKey, Databases, Google, Chemical structures, Patents, PubChem, ChemSpider

## Abstract

While chemical databases can be queried using the InChI string and InChIKey (IK) the latter was designed for open-web searching. It is becoming increasingly effective for this since more sources enhance crawling of their websites by the Googlebot and consequent IK indexing. Searchers who use Google as an adjunct to database access may be less familiar with the advantages of using the IK as explored in this review. As an example, the IK for atorvastatin retrieves ~200 low-redundancy links from a Google search in 0.3 of a second. These include most major databases and a very low false-positive rate. Results encompass less familiar but potentially useful sources and can be extended to isomer capture by using just the skeleton layer of the IK. Google Advanced Search can be used to filter large result sets. Image searching with the IK is also effective and complementary to open-web queries. Results can be particularly useful for less-common structures as exemplified by a major metabolite of atorvastatin giving only three hits. Testing also demonstrated document-to-document and document-to-database joins via structure matching. The necessary generation of an IK from chemical names can be accomplished using open tools and resources for patents, papers, abstracts or other text sources. Active global sharing of local IK-linked information can be accomplished via surfacing in open laboratory notebooks, blogs, Twitter, figshare and other routes. While information-rich chemistry (e.g. approved drugs) can exhibit swamping and redundancy effects, the much smaller IK result sets for link-poor structures become a transformative first-pass option. The IK indexing has therefore turned Google into a *de-facto* open global chemical information hub by merging links to most significant sources, including over 50 million PubChem and ChemSpider records. The simplicity, specificity and speed of matching make it a useful option for biologists or others less familiar with chemical searching. However, compared to rigorously maintained major databases, users need to be circumspect about the consistency of Google results and provenance of retrieved links. In addition, community engagement may be necessary to ameliorate possible future degradation of utility.

## Introduction

The major chemical databases now facilitate structured queries using a wide range of specifications including sketcher inputs, SD file uploads, semantic names, synonyms, IUPAC names, SMILES, InChI strings and InChIKey [1]. From the instructions and trial-and-error searches, users can become familiar with the capabilities and quirks of these interfaces, despite the lack of a common “look and feel”. Paradoxically, while users are doubtless cognizant of Google as an adjunct to conventional database searches, they may be less familiar with its capabilities and limitations. Consequently, they may either not bother, for example if database queries return approximately what they expect, or only turn to it in the later stages of an information retrieval triage. Chemical searchers are encouraged to reconsider this because the InChIKey was designed to be indexed by search engines and the potential effectiveness of InChI searching in Google was noted as early as 2004 [2].

Full details are supplied by other articles in this special issue but the InChIKey consists of the hashed connectivity information of the full InChI string [3]. The overall length is fixed at 27 characters, including the two separators. The first 14-character hash-block constitutes the skeleton inner layer. This is followed by a hyphen, then an 8-character hashed-block from the remaining layers of the InChI string. This is followed by a single character for the version, another hyphen, and a final character indicating the number of protons.

This article assesses the utilities and limitations of InChIKey searching and provides some pointers for exploitation. The term “in the wild”, as used in the title, means this article is not about implementation within databases *per se* (this is also described in other articles in this special issue) but about their utility for being found on the open web. Google is not the only significant search engine but is clearly the default choice against which others are compared and will thus be the focus of this article (from this point on InChIKey will be abbreviated to IK).

## Getting IKs into Google

There are a number of available descriptions of Google indexing [4]. Nonetheless, in the context of understanding search results, a brief outline is useful. Google’s web-crawling robot, the Googlebot, passes billions of web pages to the indexer but can also be pointed to these via active submissions. It is this process by which chemical data source website administrators can influence how much, how fast, and which parts of their data records, including the IKs, are “surfaced” in the sense of their links being included and ranked in search results. The key step is the submission of an XML Sitemap containing the selected URLs to Google Webmaster Central. Informal questioning indicates major resources such as ChemSpider and PubChem make use of this process but the extent to which smaller databases may or may not do this is unknown. Additional local modifications such as the uniqueness of metadata tags and their position on pages can enhance indexing and ranking of results. Such tweaking is termed Search Engine Optimization (SEO) but thus also refers to the less-reputable practice of commercial arrangements for boosting site rankings. There are some significant chemical information resources that either do not yet include the IK (e.g. the Therapeutic Target Database TTD) or take no active steps to enhance indexing. In the former case future incorporation would be recommended and in the latter, the amount of traffic and links on that site will determine the result ranking.

## The atorvastatin hit list

**Figure 1:**
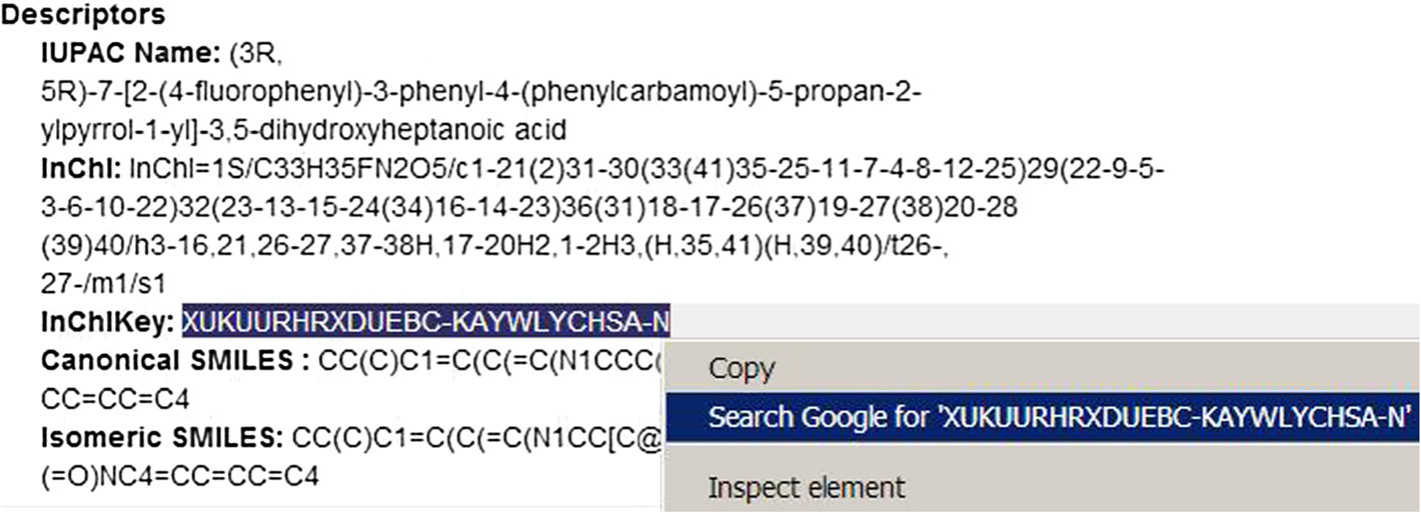
**The PubChem “Compound Information” section for atorvastatin represented in CID 60823. **The blue highlighting over the IK shows the standard web browser search launch option from the left mouse button. Note that either the inner skeleton layer or the full key string can be selected for the search.

An introductory example for searching is provided by the PubChem entry for what was the world’s best-selling prescription pharmaceutical, atorvastatin (Figure 1) and the search results for different term types and synonyms from that record have been collated (Table 1) .

**Table 1:** Search engine counts for terms related to atorvastatin (October 2012)

**Search term**	**Count**
Lipitor (Trade name) Google	35.6 million
atorvastatin (International Non-proprietary Name, INN) Google	8.9 million
“134523-00-5” (CAS number) Google	65,000
"CI-981" (Park-Davis internal code) Google	10,600
“IUPAC name”(PubChem version) Google	2,060
Canonical SMILES Google (PubChem version, with “”)	877
InChI skeleton: Google XUKUURHRXDUEBC	826
InChI skeleton: Google Images	~150
InChI skeleton: Google Scholar	1
InChI full key: Google XUKUURHRXDUEBC-KAYWLYCHSA-N	633
InChI full key: Google (nominally non-redundant subset)	201
InChI full key: Google Images	~140
InChI full key: Google Scholar	1
InChI full key: Bing	87
InChI full key: Yahoo	15

The parameter “” can be used to fix the order in the search string to improve specificity. Note the complete InChI string could not be included due to the query box restriction to 32 words, “CID 60823” gave mostly false-positives and some of the depictions in Google image results made the counting of individual renderings difficult.

**Figure 2:**
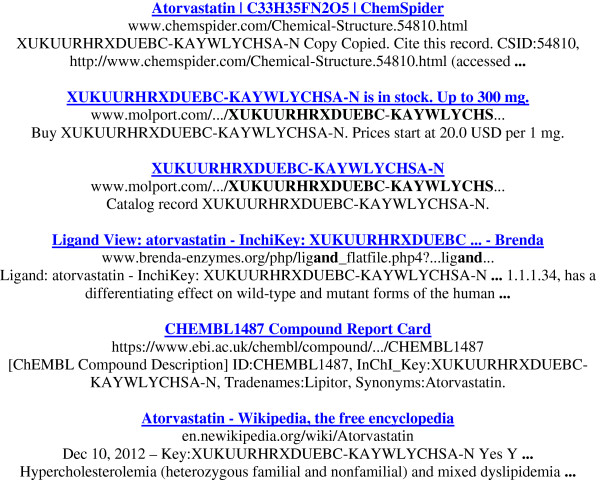
The six top-ranked hits from the atorvastatin IK (XUKUURHRXDUEBC-KAYWLYCHSA-N) in Google (October 2012).

For those interested in the non-InChI results in Table 1 they can be reproduced for inspection and/or compared to equivalent terms for other drugs. They have various utilities, including combined query options, which cannot be explored here. Not unexpectedly, the IK shows highest specificity. Note also that both the skeleton truncation search and queries against images returned informative results. The single Google Scholar hit (by full IK or skeleton) is to a publication “A novel strategy towards the atorvastatin lactone” where the authors had included a set of IKs in the text [5]. The first six entries of the IK Google returns are shown (Figure 2).

The first pages are a mixture of both the expected and the less expected. From the major sources ChemSpider was ranked 1st, ChEMBL 5th, Wikipedia 6th, PubChem 8th, DrugBank 10th, ChEBI 13th and PDB at 19th. Perhaps less expected were the chemical supplier entries at 2nd and 3rd, the BRENDA enzyme database at 4th, chemicalize.org at 13th, a publication (different to that found by Google Scholar) at 14th, an IUPHAR database entry at 31st and, at 45th a SureChem processed patent (for Wikipedia, the publication and SureChem, the IK was not on the landing page but was indexed via a link to that page). Note also that, strictly speaking, the chemical supplier entry at 2nd was a false-positives because, while the catalogue header matched correctly, the IK in the web page entry was FQCKMBLVYCEXJB-UHFFFAOYSA-N for the hemi-calcium salt (this source-specific issue is being addressed, Dr. Imants Zudans, personal communication). From the total return of 633 hits the Google “similar to” cut-off came in at 201. The heuristics of this are opaque but inspection did suggest that the remaining hits were highly redundant in a chemical information context, even if not strictly duplicates.

While the practical utility of large hit-lists for information-rich structures, such as approved drugs, might not be considered high (for the record, the aspirin IK returns 4,300) the result set does illustrate salient features. One is the ability to instantly connect across most well-established databases. While these are extensively linked between themselves, navigating between them would take an inordinate number of clicks and many are not yet instantiated as RDF to enable open data linking. Where large lists are returned, the Google advanced search filtration options can be useful [6]. For example, using exclusion options (e.g. not chemspider, chembl, wikipedia, ebi, or pubchem) cuts the list back to 211, and restricting to one year gives a single result. For positive filtration (i.e. selection), the domain origin “surechem.org” gives 54 processed patent document links and “ebi.ac.uk/” returns just 19 links to the Hinxton databases. These advanced operators can also be formulated into the standard search box rather than using the advanced search page. Note that the IK search offers what could be termed circular complementarity to database queries. This means that entering via any database link would either include the IK or link to it. A repeat search then picks up the others, regardless of which one was used as the entry point.

**Figure 3:**
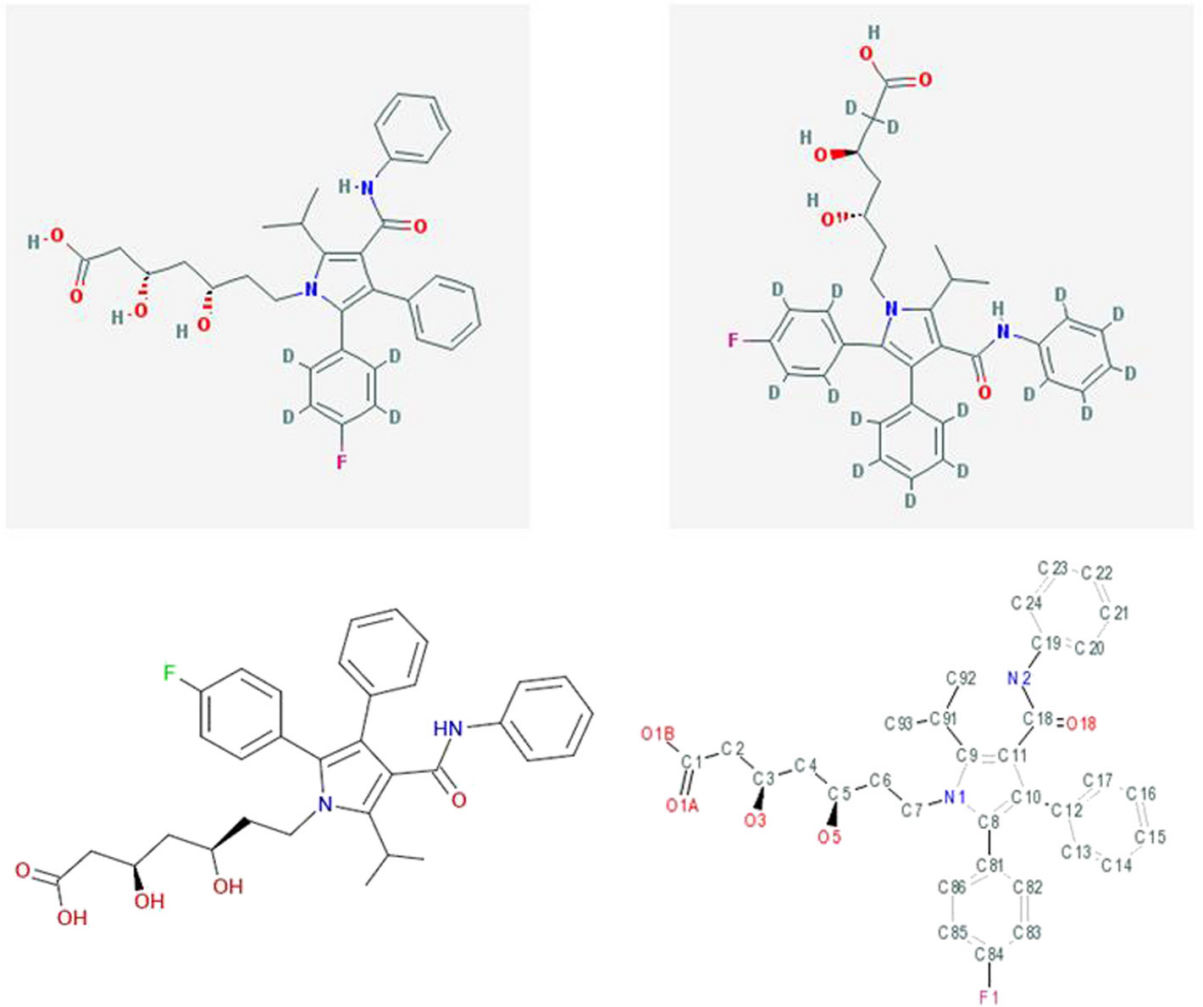
**Selected matches of the atorvastatin skeleton key XUKUURHRXDUEBC in Google Images. **The links for the images were PubChem CIDs 25145244 and CID 53233924, both deuterated (top row) with CHEMBL1487 and PDBeChem117 (bottom row).

With any search engine results the questions arise as to how many pages users are prepared to inspect or pursue off-line processing (the latter is made difficult by Google but there are some technical options that will not be covered here). For a typical IK result set, inspection of multiple pages up to the Google cut-off may not be that onerous (e.g. QGJUIPDUBHWZPV-SGTAVMJGSA-N for saxagliptin has only seven pages) and, as specified above, filtration can be used to cut down the results. The different utility of searching Google images with IKs is shown below, in this case for a skeleton key (Figure 3).

The grey-boxed renderings of first two images are distinctive for the PubChem entries that happen to be deuterated. These are followed by two from PDBe. Notably, the rankings between the Web and Images results are different. While images and their associated links defy any simple classification of true positives or result counts, the immediate visual rendering of structures may be intuitively preferable as an exploratory search and a recent Google enhancement now renders a higher quality image and description of the link by clicking on the thumbnails. In addition, because the skeleton query indicates the presence of isomers, this may be the most efficient first-pass. The image list also highlights the IK mediated detection of what could be termed “boutique” resources. By this is meant simply that they are less familiar than major databases and may not only offer unique features but also would be difficult to find any other way. An example on the first page of images from the skeleton search was BioPath. This has been published but is less well known than other pathway resources [7].

**Figure 4:**
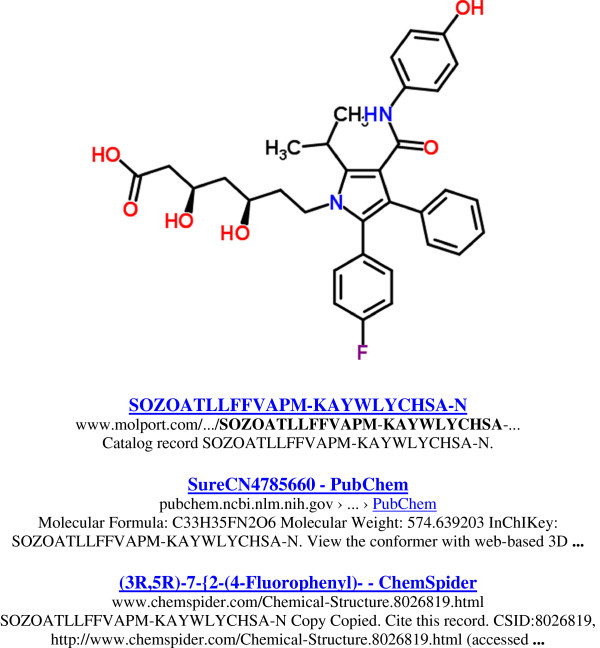
**Search of the metabolite para-hydroxy atorvastatin. **The ChemSpider structure (CS 8026819) is shown (top panel). The IK Google search gives only three matches (lower panel).

Perhaps a more convincing demonstration of practical value is when IK searching connects to structures less frequently captured by web indexing. As an example, we can choose one of the major metabolites of atorvastatin (Figure 4) .

The difference to the precursor results (i.e. 633 vs. three) is striking, especially considering 70% of the *in vivo* pharmacological activity is attributable to just two metabolites [8]. We can generate analogous results (not shown) for the second major metabolite, para-hydroxy atorvastatin, CZBPKFICAYVHHM-JWQCQUIFSA-N. This gives 19 links but the majority of these are derived, directly or indirectly, from the PDB ligand entry for this structure. Truncating back to the ortho derivative skeleton, SOZOATLLFFVAPM, expands the list from three to nine and brings in ChemSpider and PubChem entries for various isomers, including the radiolabeled [2H5]- p-hydroxyatorvastatin (CID 16053351). Analogously, the para skeleton, CZBPKFICAYVHHM, expands the list to 35, but this is redundancy-trimmed by Google to 21. Unfortunately, the top-ranked ranked match includes one of the few complete false-positives found in this assessment, where DrugBank returns a “No results found”.

The IK has another intrinsic property that is both a disadvantage and an advantage. Unlike SMILES, InChI strings or IUPAC names, they cannot generate a structure algorithmically but only link to it via a look-up operation. One of the consequent advantages is the provision of *de facto* secure searching in the sense that an IK will only have a Google match if the structure is public (although technically, the surfaced IK need not actually be openly linked to a structure). The ability to check nominally proprietary (e.g. internally-designed) structures in this way is unlikely to be relied upon by companies with sufficient resources to not only license major commercial databases but also maintain an internal (licensed) ChemSpider version as well as updated PubChem downloads [9]. Nevertheless, drug discovery teams with more limited resources at least have the alternative of IK “blinded” (and free) novelty checking. Use of this option is also likely to increase because commercial databases can no longer rely on being able to subsume all prior-art chemical structural data indexed by Google.6

## External Google vs. internal structured databases IK searches

**Figure 5:**
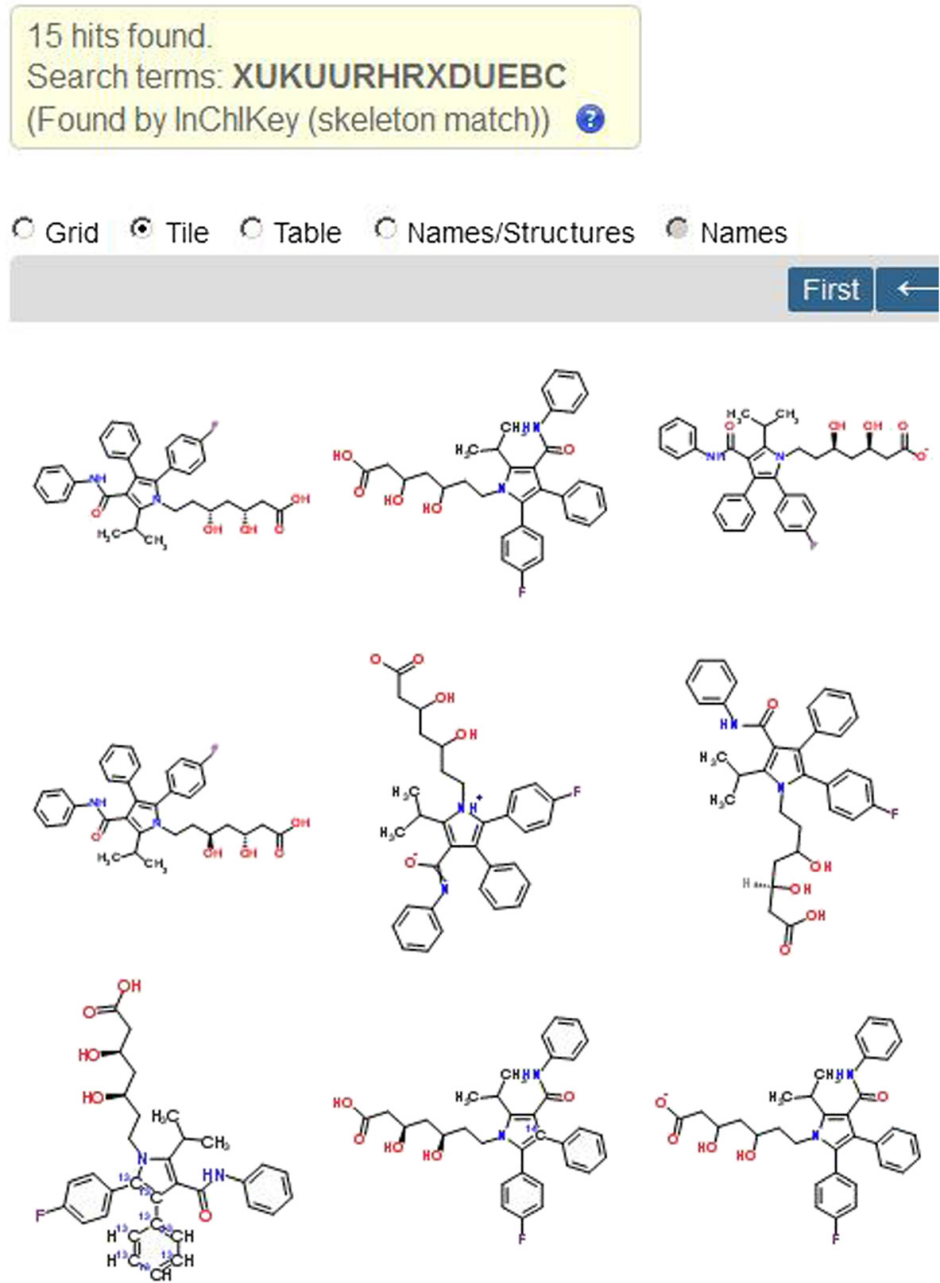
Results from the “Compounds with the same skeleton” internal search of the atorvastatin skeleton key from the ChemSpider interface (nine out of the 15 displayed hits are shown).

To give an idea of the relative utility we can compare internal database and external results from the same IK searches. Both alternatives are implemented in the ChemSpider search interface (Figure 5).

As we can see (Figure 5) the 15 internal results bring back more structures that corresponding Google Images search (Figure 3). While the Google filtered search ("XUKUURHRXDUEBC" site: http://www.chemspider.com/) returns 22 matches, only 15 pass the Google redundancy cut-off. Thus, for ChemSpider the internal or external searches gave the same result. Further investigations uncovered differences in internal-vs.-external IK search behavior between databases. For example, the ChemSpider and PubChem interfaces, as expected, will both return unique full IK matches (although search times are longer than in Google). However, PubChem cannot execute a truncated or wild-card IK search (but imminent Entrez enhancements will enable this, Dr. Evan Bolton, personal communication). Both full and skeleton searches can be used in the ChEBI interface but, currently, neither in ChEMBL. Thus, the Google IK search can return results that currently have no direct database-internal equivalents. On the other hand, attempting to resolve salt forms comes up against the inherent disadvantage of the IK in that mixtures do not have distinct layers for the com ponents. Thus, the PubChem entry for atorvastatin (CID 60823) needs to be connected internally (via the “Related Data” option) to 151 mixture CIDs that each have a unique IK. Despite this disadvantage, the ability to connect to these indirectly via IK searching, is still possible because PubChem splits every mixture into component CIDs which, in turn, become Google–indexed IKs.

## Document-to-database connections

**Figure 6:**
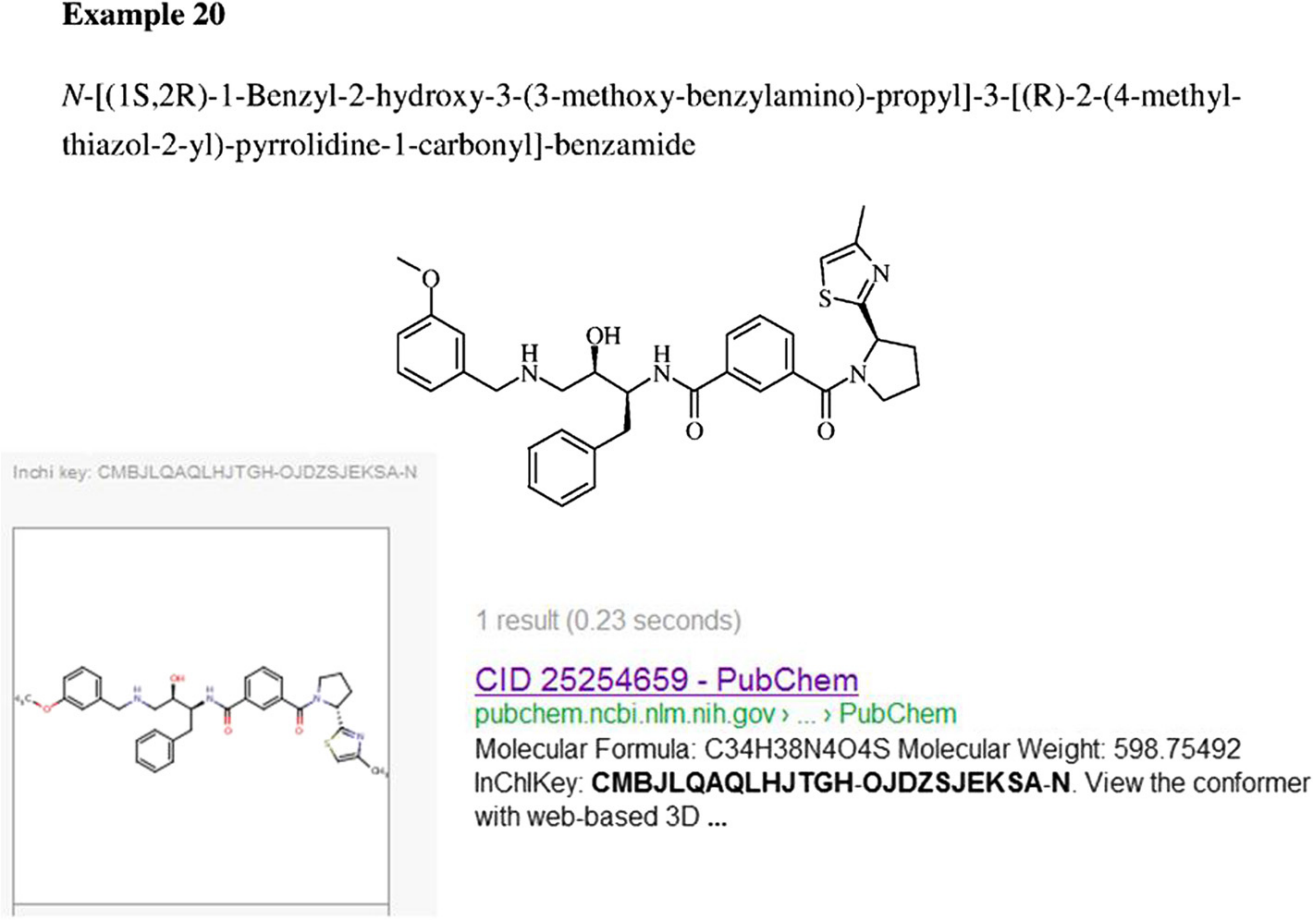
**Search results of example 20 from the SureChemOpen extraction of WO2012028563. **The depiction in the original PDF is shown at the top. The SureChemOpen structure conversion, including the generated IK, is lower right and the single Google match for that IK lower left.

Databases increasingly provide cross-references between chemical records, patent documents, PubMed abstracts and a small proportion of full-text papers. However, there are still many millions of compounds in various text forms that are not captured. In such cases, the IK search has unique capability to facilitate document-to-database joins. While this requires the generation of an IK from whatever primary representational form is used in the text there are a number of open tools and resources that either store pre-calculated conversions or compute them on-the-fly. The largest of these is SureChemOpen that includes ~12 million structures, each with the corresponding IK linked to the position in the patent documents they were automatically extracted from [10]. As an example, searching this resource with the gene name Beta Amyloid Cleaving Enzyme 2 (BACE2 a diabetes drug target) retrieves WO2012028563. Selecting an embedded image for one of the exemplified structures opens up a rendering and properties record from which an IK search can be launched directly as shown in Figure 6.

Performing this operation from within the patent thus answers the question “is this a known structure?” simply via the browser highlight and right click, compared to performing multiple database checks. In this case (Figure 6) there is just one match to CID 56846820, but this not only connects to five PubChem sources but also has 321 structurally similar neighbors (details can be explored in the PubChem links. Note that this initial connectivity, enabled via the IK, can be exploited reciprocally. For example, a different structure selected from a nearest-neighbour (CID 59570194) can be searched back against SureChemOpen (but via canonical SMILES not IK) and connects to another patent WO2009015369.

**Figure 7:**
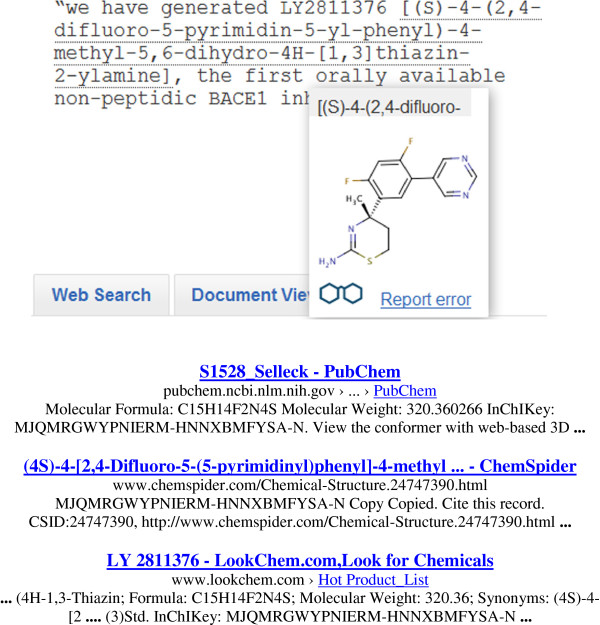
**Conversion of an IUPAC name into an IK for Google searching. **The underlined text from PubMed 22090477 (upper panel) has been converted to a structure including the generated IK at chemicalize.org. The three Google matches to this IK are shown (lower panel).

The theme of connecting between structures specified in documents was extended in PubMed by executing a search for Beta Amyloid Cleaving Enzyme 1 (BACE1, an Alzheimer’s disease drug target in this case) plus the term “inhibitor”. One of the recent returns was a 2011 abstract (PMID 22090477) describing a discontinued clinical candidate [11]. The important point here is that the structure is only instantiated as an IUPAC name in the text source, juxtaposed with (i.e. mapped to) the company code LY2811376. In such cases, open resources such as the Open Parser for Systematic IUPAC Nomenclature (OPSIN) [12], the Chemical Identifier Resolver [13] or chemicalize.org [14] can be used to generate structures from IUPAC names. As an example the generation of an IK using the latter is shown in Figure 7.

The results (Figure 7) show that processing the IUPAC name in the abstract text and generating the IK enables the Google search to match two major database entries and a chemical vendor. There are some interesting corollaries to the utility of these connections. The first is, despite the structure being connected to eight different sources in CID 44251605, the code name LY2811376 has no synonym link to a PubChem entry, even though MeSH has indexed the IUPAC name as a supplementary concept. Therefore, the IK search has not only facilitated this code name-to-structure mapping but this was also independently corroborated by a vendor entry (the last Google result in Figure 7). The second point is that the structure can now be mapped back to a different BACE1 patent, US20090275566, not only via SCRIPDB in PubChem (SID 137986191) but also to the whole patent family via SureChemOpen (SID 153233601

Regardless of how IK searching may be combined with other resources it enables document-to-structure-to-database-to-document mapping workflows that can establish joins between patents, papers, abstracts and databases. Many of these are difficult to connect otherwise (particularly from patents “back” to databases). Note also that the constraint of IK-to-IK exact matching can be circumvented because they co-exist with SMILES and InChI strings for conversion tools and database records. This enables chemical similarity to be explored and extended in parallel with IK connectivity.

## Sharing

The IK can expedite the finding by others of structures researchers are engaging with beyond their local team of collaborators. This can take many forms but “Open Notebook Science” is perhaps the most radical in offering the shortest route for structures, being designed, made and characterized experimentally, to be concomitantly surfaced in close to real time. (e.g. [15]) While open lab books currently specify structures mainly as IUPAC names, the text is indexed and IK inclusion will become more widespread via automation (Dr. Matthew Todd, personal communication). It is clearly in the communal interest, not only for novel bioactive chemistry but also new data linked to extant structures, to eventually flow into major public repositories, as has been recommended in the Minimum information about a bioactive entity (MIABE) guidelines for publication [16]. However, the simple inclusion of an IK in an open report indexed by Google could precede this by many months. There are other ways for individuals or small teams to enable their IK and associated data to become searchable. The fastest of these is any posting in the Google Blogger application where the indexing is instantaneous [17]. Twitter IK postings also become visible but this seems to depend on aggregation via secondary portals. Testing with Dropbox and figshare indicated IKs were not currently being picked up from the former but were surfacing from data sheets deposited in the latter [18]. As open-source drug discovery (OSDD) expands new sharing options will doubtless be explored. A more pragmatic form of sharing is the addition of out-links for chemistry specified in, for example, an internal report, full-text journal paper or patent. The inclusion (or later addition) of an IK not only facilitates enrichment of the document by manual expert mark-up with selected source links chosen from the initial Google result set, but also provides an instant update and/or source expansion during reading by simply refreshing the search.

## Conclusions and outlook

The fact that IK Google searches generate a comprehensive set of resource links in ~0.3 seconds with a low false-positive rate and includes the skeleton key option of isomer expansion, demonstrates not only that this has become an important search option but is also one of the major successes of the InChI Trust. While it is arguably less valuable for common chemistry, the utility for link-poor structures (i.e. a couple of pages of results or less) is transformative because it turns Google into the *de-facto* largest open global chemical information source. Thus, the effective merging of 47 million PubChem entries with the 28 million from ChemSpider now presents the one-stop search that users have been waiting for because of the unknown proportions of content unique to each (the current time lag for IK indexing of new PubChem entries is being addressed, Dr. Evan Bolton, personal communication). Notably, this expanding “IK space” will encompass the majority of patent-extractable chemistry already amounting to ~14 million entries in PubChem. A final utility worth specifying is derived from a combination of the conceptual simplicity of string matching, the high specificity of results, general familiarity with Google, and prominent display of source names in results. This means that those less experienced with chemical structure queries (e.g. Bioinformatitians, Biologists, Pharmacologists and Information Scientists) should find IK searching easy to exploit.

We should take nothing away from Google for the utilities demonstrated here. However, users of the IK (or any other technical search term) do need to be circumspect. Although major databases would never claim perfection they devote much effort towards maintaining stability, chemistry rules, search reproducibility, updating and source provenance (at least in the secondary sense where primary provenance and quality ultimately resides with the submitters). As a chemical search engine Google cannot ensure any of this and its behavior can be profoundly quirky. The most disconcerting is the “shifting sand” effect where result numbers and link rankings change significantly over time scales as short as weeks. While this can be due to many factors such as algorithmic tweaking on the Google side or page availability and SEO changes on the source side, the process is largely opaque. The provenance problem presents itself in the difficulty of discriminating between links to potentially valuable boutique resources on the one hand and those that have become dangerously outdated from under-resourcing on the other (but still get ranked). Another example of capricious behavior was recorded during the course of this work when a question was posted to the BioStar Q&A site including the atorvastatin IK. Unwittingly, this link is currently ranked highly in the Google search results. Another caveat to be aware of is “swamping”. This was encountered in the atorvastatin result set where, from approximately the 80th position onwards, the “dark side” of pharmaceutical suppliers appeared for ~30 results, including tricks such as multiple domain name spawning. A related chemical term “swamping” problem has reported in recent exercises to retrieve structures associated with company code names [19]. Because of multiple replications of clinical trial information and press announcement recycling, unfiltered Google searching with drug code names used in clinical trials has become effectively useless.

Notwithstanding the achievements of Google Scholar, the standard Google web search cannot be expected to “look after” result quality and specificity in the way we take for granted from our collective feedback to chemical database staff and curation teams. Consequently, there may be future trends or areas for concern that we, as the user community, may seek to ameliorate (assuming we are in position to influence such matters). To take a purely hypothetical example, if the PubMed/MeSH system (and/or the majority of publishers) decided to append IKs to each of the over 5000 abstracts mentioning atorvastatin, this could lead to “swamping” (only a few appear in the current atorvastatin IK results). The dilemma here is that this same option becomes a major benefit if it were implemented only for rarer structures (e.g. LY2811376 mentioned above). This could be influenced by author choice, for example, IK inclusion in the abstract should make this retrievable in Google Scholar.

## Competing interests

The author is on the Advisory Board of SureChem whose Open database was used for some examples.
